# 超高效液相色谱-二极管阵列检测法同时测定化妆品中22种防晒剂

**DOI:** 10.3724/SP.J.1123.2020.12001

**Published:** 2021-04-08

**Authors:** Keming ZHANG, Ming DENG, Yuanyuan SU, Dong XIE, Youcheng XU, Xianghong LIU

**Affiliations:** 1.广西食品药品检验所, 广西 南宁 530021; 1. Guangxi Institute for Food and Drug Control, Nanning 530021, China; 2.桂林医学院, 广西 桂林 541004; 2. Guilin Medical University, Guilin 541004, China

**Keywords:** 超高效液相色谱, 防晒剂, 紫外吸收剂, 化妆品, ultra-high performance liquid chromatography (UHPLC), sunscreen agents, ultraviolet (UV) filters, cosmetics

## Abstract

建立了一种超高效液相色谱-二极管阵列检测法同时测定不同种类化妆品中22种防晒剂的方法。样品首先加入少量四氢呋喃,涡旋、分散、混匀(蜡质化妆品可于50 ℃超声,以便分散完全),然后加入0.1%(v/v)甲酸乙醇溶液振摇、超声提取,稀释、过滤后经Poroshell 120 EC-C_18_(100 mm×4.6 mm, 2.7 μm)分离,乙腈-异丙醇-50 mmol/L乙酸铵水溶液(含0.05%(v/v)甲酸)梯度洗脱,二极管阵列检测器检测,检测波长311 nm,外标法定量。结果表明,22种防晒剂在各自的范围内线性关系良好,相关系数(*r*)均在0.998以上。选取两种不同基质类型的样品,在低、中、高3个不同添加水平下的平均回收率为85.2%~112.3%,相对标准偏差(RSD)在0.5%~6.5%之间,方法的检出限和定量限分别为0.7~64 mg/kg和2.4~215 mg/kg。在2、10、50 mg/L3个水平的混合标准溶液的稳定性试验中,甲酚曲唑三硅氧烷组分12 h内稳定,其余组分36 h内稳定。采用该方法测定市售16份化妆品中的防晒剂,其中5份样品检测结果与厂家提供的配方值匹配良好。与《化妆品安全技术规范》(2015年版)收载方法相比,该方法在流动相组成和提取溶剂选择方面,摒弃了四氢呋喃和高氯酸等腐蚀性溶剂的使用,改善了弱极性组分的提取效率,同时增加了检测组分二苯酮-2,被测物稳定性更好,实验操作更便利高效,分离效果更好,结果更准确,兼顾绿色环保,可用于化妆品防晒剂的日常检测。

## Abstract (en)

A fast and simple ultra-high-performance liquid chromatography using diode array detector (UHPLC-DAD) technique has been developed for the simultaneous determination of 22 sunscreen agents (UV filters), viz. phenylbenzimidazole sulfonic acid (PBS), terephthalydene dicamphor sulfonic acid (TDS), benzophenone-4 (BZ4), camphor benzalkonium methosulfate (CBM), benzophenone-2 (BZ2), benzylidene camphor sulfonic acid (BCS), benzophenone-3 (BZ3), 3-benzylidene camphor (3BC), isoamyl *p*-methoxycinnamate (IMC), 4-methylbenzylidene camphor (MBC), diethylamino hydroxybenzoyl hexyl benzoate (DHHB), octocrylene (OCR), butyl methoxydibenzoyl methane (BDM), ethylhexyl dimethyl PABA (EDP), ethylhexyl methoxycinnamate (EMC), homosalate (HS), ethylhexyl salicylate (ES), diethylhexyl butamido triazone (DBT), ethylhexyl triazone (ET), drometrizole trisiloxane (DRT), methylene bis-benzotriazolyl tetramethylbutylphenol (MBP), and bis-ethylhexyloxyphenol methoxyphenyl triazine (EMT) in cosmetic products. Different parameters, such as column type, oven temperature, mobile phase composition, and detection wavelength, were studied. The best chromatographic separation was obtained under the following conditions: Poroshell 120 EC-C_18_ (100 mm×4.6 mm, 2.7 μm) column set at 25 ℃ and gradient acetonitrile-isopropanol-water (containing 50 mmol/L ammonium acetate and 0.05% (v/v) formic acid) as the mobile phase, pumped at a flow rate of 0.5 mL/min, with a wavelength of 311 nm. The proposed UHPLC-DAD technique provided separation of the 22 target sunscreen agents within 35 min, with the optimized sample pretreatment procedure below. First, samples were mixed thoroughly by adding 2 mL or 5 mL tetrahydrofuran, followed by vortex and dispersal. If the wax samples were still not homogenized completely, an ultrasonic dispersal protocol with heating to 50 ℃ was adopted. Second, the homogeneous samples were ultrasonically extracted with ethanol containing 0.1% (v/v) formic acid. The developed method showed good linear relationships, with correlation coefficients of no less than 0.998. Two kinds of samples with different matrix types were fortified at three levels. The average spiked recoveries of 22 UV filters ranged from 85.2% to 112.3%, with the relative standard deviations (RSDs) ranging from 0.5% to 6.5%. The limits of detection were between 0.7 and 64 mg/kg, and the limits of quantification ranged from 2.4 mg/kg to 215 mg/kg. Moreover, the stabilities of the mixed standard solutions at the levels of 2, 10, and 50 mg/L were tested. The stability results showed that drometrizole trisiloxane was stable for 12 h, while the others were stable for 36 h. The reliability of the developed method was demonstrated by applying it to 16 commercial sunscreen-containing cosmetic samples obtained from the Chinese market. The levels determined in this study agreed well with those of five commercial samples (such as emulsion and cream). The method developed was remarkably different from the standard method, which is mentioned in the Safety and Technical Standards for Cosmetics (2015 edition), especially in terms of mobile phase composition and extraction solvent. Compared to the standard method, this method bypassed the use of large amounts of corrosive solvents like tetrahydrofuran and perchloric acid, thus improving the extraction efficiency of low-polarity components like drometrizole trisiloxane, methylene bis-benzotriazolyl tetramethylbutylphenol, and bis-ethylhexyloxyphenol methoxyphenyl triazine, and the analytes were well separated with better stability. Benzophenone-2 was added to this method as another detection component. The good analytical features, as well as their environment-friendly characteristics, make the presented method suitable not only for routine analysis in cosmetics industries, but also as a candidate reference method for sunscreen analysis.

随着护肤知识的普及,防晒日渐受到消费者重视,防晒剂广泛应用于各类化妆品中,包括防晒霜、防晒乳、防晒喷雾、隔离霜、唇膏以及粉饼等,用量通常在0.1%~10%之间。由于防晒类化妆品中的防晒成分有一定的副作用,世界各国对防晒剂种类及最大使用浓度有不同要求^[1,2]^。我国《化妆品安全技术规范》(2015年版)^[3]^(下文简称《规范》)收载了27种准用防晒剂,包括25种化学防晒剂和2种物理防晒剂。各化学防晒剂的使用频率有较大区别,根据2020年本实验室收集的121批防晒样品的实验结果统计,甲氧基肉桂酸乙基己酯使用频率最高(86.0%),其次为双-乙基己氧苯酚甲氧苯基三嗪(50.4%)、二乙氨羟苯甲酰基苯甲酸己酯(40.5%)、水杨酸乙基己酯(37.9%)、丁基甲氧基二苯甲酰基甲烷(36.6%)等14种组分,苯基二苯并咪唑四磺酸酯二钠、二甲基PABA乙基己酯、樟脑苯扎铵甲基硫酸盐、亚苄基樟脑磺酸、二苯酮-4/二苯酮-5、3-亚苄基樟脑等6种组分未见使用。

目前,防晒剂的检测方法有高效液相色谱法^[4,5,6,7,8,9,10,11]^、超高效合相色谱法^[12,13]^、液相色谱-质谱联用法^[14,15,16,17]^、气相色谱-质谱联用法^[18,19]^等,其中高效液相色谱法应用最广。《规范》收载了5种检测方法,2019年又新增了22种防晒剂的检测方法^[20]^。其中新增的22种防晒剂的检测方法操作最便捷,但仍存在诸多不足:流动相四氢呋喃含量高,易腐蚀液相色谱聚醚醚酮(PEEK)管路,且污染环境;被测组分中甲酚曲唑三硅氧烷的稳定性差,大批量测定数据失真;提取溶剂兼容性差,弱极性组分提取不完全等。因此,本研究选取霜、乳、水、喷雾、唇膏等不同类型基质进行研究,对提取溶剂进行了改进;对分析过程中采用的流动相种类及改性剂进行了优化,使基线平稳。在系统优化的基础上,采用小粒径(2.7 μm)的C_18_超高压色谱柱,实现了全部22种组分(包括《规范》未收录的二苯酮-2)的基线分离。方法简便快捷、结果准确、环保低毒,具有更强的适用性和耐用性。

## 1 实验部分

### 1.1 仪器、试剂与材料

UltiMate 3000高效液相色谱仪(美国Thermo Scientific公司),配置DAD检测器;Milli-Q超纯水仪(美国Millipore公司); G560E快速混匀器(美国Scientific Industries有限公司); S120H超声波发生器(德国Elma公司); XS205DU电子天平(瑞士梅特勒公司)。

甲醇、乙腈、异丙醇、四氢呋喃、乙酸铵、甲酸均为色谱纯(美国Fisher公司);水为超纯水。

22种防晒剂标准品及纯度为苯基苯并咪唑磺酸(PBS)96.2%、二苯酮-4(BZ4)96.2%、4-甲基苄亚基樟脑(MBC)99.83%、二乙氨羟苯甲酰基苯甲酸己酯(DHHB)99.4%、二甲基PABA乙基己酯(EDP)99.2%、水杨酸乙基己酯(ES)99.4%、胡莫柳酯(HS)98.86%、亚甲基双-苯并三唑基四甲基丁基酚(MBP)97%(购自Dr. Ehrenstorfer公司),亚苄基樟脑磺酸(BCS)98.9%、二苯酮-3(BZ3)100%、对甲氧基肉桂酸异戊酯(IMC)99.2%、奥克立林(OCR)99.6%、丁基甲氧基二苯甲酰基甲烷(BDM)99.7%、甲氧基肉桂酸乙基己酯(EMC)99.5%、二乙基己基丁酰胺基三嗪酮(DBT)98.3%、乙基己基三嗪酮(ET)94.6%、双-乙基己氧苯酚甲氧苯基三嗪(EMT)98.7%(购自ANPEL公司),对苯二亚甲基二樟脑磺酸(TDS)98.0%(购自International Laboratory公司),樟脑苯扎铵甲基硫酸盐(CBM)98.0%、3-亚苄基樟脑(3BC)97.0%(购自Sigma-Aldrich公司),二苯酮-2(BZ2)95.0%(购自CNW公司),甲酚曲唑三硅氧烷(DRT)94.4%(购自CATO公司)。

16批样品分别由厂家提供或购于本地商场、超市。

### 1.2 标准溶液的配制

精密称取苯基苯并咪唑磺酸标准品50 mg至10 mL棕色容量瓶中,先加入1.0 mL甲醇,然后滴加1.0 mol/L氢氧化钠溶液至溶解,用甲醇定容至刻度,摇匀,即得苯基苯并咪唑磺酸标准储备液,置于4 ℃冰箱备用。

分别精密称取丁基甲氧基二苯甲酰基甲烷、乙基己基三嗪酮、亚甲基双-苯并三唑基四甲基丁基酚和双-乙基己氧苯酚甲氧苯基三嗪标准品各50 mg至不同的10 mL棕色容量瓶中,先加入4.0 mL四氢呋喃,超声至溶解,用甲醇定容至刻度,摇匀,即得上述4种防晒剂的标准储备液,置于4 ℃冰箱备用。

精密称取其余17种防晒剂各50 mg至不同的10 mL棕色容量瓶中,用甲醇溶解并定容至刻度,摇匀,即得其余17种防晒剂的标准储备液,置于4 ℃冰箱备用。

混合标准储备液:根据各防晒剂使用限量及色谱响应差别,分别移取各标准储备液适量,用甲醇稀释成100 mg/L的混合标准储备液,置于4 ℃冰箱备用。

混合标准工作溶液:用甲醇将混合标准储备液逐级稀释成1、2、5、20、50 mg/L的系列标准工作溶液,置于4 ℃冰箱备用。

### 1.3 样品前处理

霜、乳、粉饼:称取样品0.25 g,置于25 mL具塞比色管中,加入四氢呋喃2.0 mL,涡旋1 min使样品分散均匀,然后用0.1%(v/v)甲酸乙醇溶液定容至刻度,摇匀,超声提取20 min,放冷至室温,准确吸取1.00 mL至10 mL具塞试管中,0.1%(v/v)甲酸乙醇溶液定容至刻度,混匀,经0.45 μm聚四氟乙烯(PTFE)微孔过滤后进行测定。

露、爽肤水、喷雾剂:称取样品0.25 g,置于25 mL具塞比色管中,0.1%(v/v)甲酸乙醇溶液定容至刻度,摇匀,准确吸取1.00 mL至10 mL具塞试管中,0.1%(v/v)甲酸乙醇溶液定容至刻度,混匀,经0.45 μm PTFE微孔过滤后进行测定。

唇膏:称取样品0.25 g,置于25 mL具塞比色管中,加入四氢呋喃5.0 mL,涡旋,50 ℃超声20 min使样品充分溶散,放冷至室温,0.1%(v/v)甲酸乙醇溶液定容至刻度,摇匀,准确吸取1.00 mL至10 mL具塞试管中,0.1%(v/v)甲酸乙醇溶液定容至刻度,混匀,经0.45 μm PTFE微孔过滤后进行测定。

### 1.4 色谱条件

色谱柱:Agilent Poroshell 120 EC-C_18_ Column(100 mm×4.6 mm, 2.7 μm);流动相:A为50 mmol/L乙酸铵水溶液(含0.05%(v/v)甲酸), B为乙腈,C为异丙醇。梯度洗脱程序:0~5 min, 80%A~50%A, 10%B~25%B, 10%C~25%C; 5~8 min, 50%A~30%A, 25%B~42%B, 25%C~28%C; 8~22 min, 30%A~25%A, 42%B~45%B, 28%C~30%C; 22~26 min, 25%A~0%A, 45%B~50%B, 30%C~50%C; 26~33 min, 0%A, 50%B, 50%C; 33~35 min, 0%A~80%A, 50%B~10%B, 50%C~10%C; 35~38 min, 80%A, 10%B, 10%C。流速:0.5 mL/min;进样量:5 μL;检测波长:311 nm;柱温:25 ℃。

## 2 结果与讨论

### 2.1 色谱条件的优化

2.1.1 色谱柱及柱温的选择

本研究考察了Luna C_18_(150 mm×2.0 mm, 3 μm)、Poroshell 120 EC-C_18_(100 mm×4.6 mm, 2.7 μm)、CSH C_18_(150 mm×3.0 mm, 1.7 μm)以及Zorbax Eclipse plus RRHD C_18_(100 mm×2.1 mm, 1.8 μm)4种型号的色谱柱对22种化学防晒剂的分离效果。结果发现CSH C_18_和RRHD C_18_均不能有效分离BZ2-BCS和OCR-BDM两组成分,Luna C_18_和RRHD C_18_均不能有效分离ET-DRT和MBP-EMT两组成分,同时色谱信号响应较低。故本实验选择Poroshell 120 EC-C_18_色谱柱,其分离得到的色谱峰响应相对较高,且分离度良好。

考察了25、30、40、50、60 ℃条件下色谱分离的情况。随着温度升高,色谱分离时间缩短,但几组物质(CBM、BZ2和BCS, 3BC和IMC, ET和DRT, MBP和EMT)分离度越来越差,BDM呈现大宽峰,峰形前延严重,不易定性定量。因此,设置恒定温度25 ℃。

2.1.2 流动相的选择

《规范》^[3,20]^选择了高浓度的四氢呋喃作为流动相。四氢呋喃易挥发,毒性较强,同时易腐蚀PEEK管路,缩短仪器使用寿命。因此,本研究考虑采用常规的甲醇或乙腈和乙酸铵水溶液添加一定比例的酸性改性剂作为流动相,以实现22种防晒剂的分离。仅采用乙酸铵-甲醇或乙酸铵-乙腈均不能实现大部分化合物的有效分离。乙酸铵-乙腈体系中,加入一定比例的异丙醇,能有效分离大部分脂溶性防晒剂。为了改善目标物的色谱峰形及分离度,往乙酸铵中加入0.05%(v/v)甲酸能有效提高DBT等化合物的响应值,同时改善OCR-BDM-EDP-EMC和CBM-BZ2-BCS的分离度。故本实验选择50 mmol/L乙酸铵水溶液(含0.05%(v/v)甲酸)-乙腈-异丙醇作为流动相,22种防晒剂的分离效果更好,信号强度相对更高。

随后优化了梯度洗脱条件。经过反复试验,改变不同时间段流动相中有机相和水相的比例,最终确定了最佳梯度洗脱条件,获得了22种防晒剂的最佳分离效果,色谱图见图1。

**图 1:**
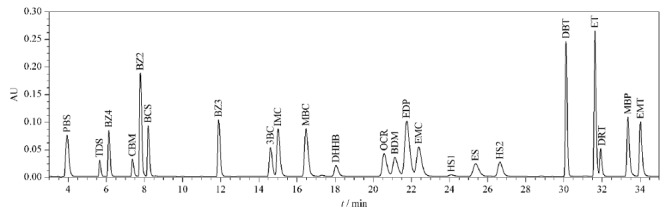
22种防晒剂混合标准溶液的液相色谱图(311 nm)

2.1.3 检测波长的确定

混合标准溶液经二极管阵列检测器在190~400 nm波长范围内全扫描,结果发现,22种目标物最大紫外吸收波长分布在4个区域。PBS、IMC、MBC、OCR、EDP、EMC、ES、HS、DBT、ET、DRT、MBP和EMT均在311 nm波长附近有最大紫外吸收;BZ4、CBM、BCS、BZ3和3BC最大吸收波长为290 nm; TDS和BZ2为340 nm; DHHB和BDM则为358 nm。《规范》所涉及方法的检测波长统一为311 nm。本方法在311 nm波长下的检测灵敏度均高于原标准。因此,为方便结果比较,选择311 nm作为检测波长对上述22种防晒剂进行定性定量分析。

### 2.2 样品前处理条件的优化

选取含有多种化学防晒剂的霜、乳类样品进行提取方法考察。根据化合物极性相似相溶的原理,我们分别考察了甲醇(极性6.6,黏度0.6)、乙腈(极性6.2,黏度0.37)、异丙醇(极性4.3,黏度2.37)和乙醇(极性4.3,黏度1.2)等溶剂代替四氢呋喃(极性4.2,黏度0.55)提取样品的效果。同时,为了抑制因苯基苯并咪唑磺酸等组分电离而增加的水溶性,向提取溶剂中加入0.1%(v/v)甲酸,增加有机溶剂提取效率。与四氢呋喃-甲醇-水-高氯酸(450:250:300:0.25, v/v/v/v)溶剂系统(标准方法^[20]^)比较,各溶剂提取效果见表1。

**表 1:** 不同溶剂提取霜、乳类化妆品中防晒剂的结果(*n*=2)

Analyte	Formula value/%	Contents in cream/%							Contents in emulsion/%						
		A	B	C	D	E	F		A	B		C	D	E	F
PBS	3.00	2.95	2.79	2.54	2.90	3.02	2.96		ND	ND		ND	ND	ND	ND
TDS	9.50	ND	ND	ND	ND	ND	ND		9.63	6.46		8.36	9.15	9.30	9.52
BZ3	4.00	4.09	4.06	4.04	4.04	4.07	4.00		ND	ND		ND	ND	ND	ND
IMC	3.00	3.24	3.09	3.15	3.09	3.14	3.10		ND	ND		ND	ND	ND	ND
MBC	2.00	ND	ND	ND	ND	ND	ND		2.02	2.04		2.09	2.09	2.09	2.04
DHHB	3.50	3.45	3.32	3.33	3.35	3.41	3.51		ND	ND		ND	ND	ND	ND
OCR	0.15	ND	ND	ND	ND	ND	ND		0.17	0.17		0.16	0.16	0.17	0.16
BDM	0.070	ND	ND	ND	ND	ND	ND		0.070	0.070		0.067	0.068	0.067	0.069
EMC	8.00	7.98	7.77	7.89	7.94	8.09	8.06		ND	ND		ND	ND	ND	ND
ES	2.00	1.92	1.89	1.67	1.95	1.99	2.08		ND	ND		ND	ND	ND	ND
HS	0.030	ND	ND	ND	ND	ND	ND		0.027	0.027		0.028	0.029	0.030	0.028
ET	1.00	1.05	1.00	1.04	1.04	1.06	0.85		ND	ND		ND	ND	ND	ND
DRT	3.00	ND	ND	ND	ND	ND	ND		3.08	3.05		3.09	3.01	3.08	2.06
MBP	3.00	0.47	0.91	2.77	2.50	2.95	0.46		0.99^*^	1.79^*^		3.58^*^	2.11^*^	4.08^*^	0.73^*^
EMT	2.50	2.39	2.51	2.52	2.54	2.53	0.91		ND	ND		ND	ND	ND	ND

Extration solvent A: methanol containing 0.1% (v/v) formic acid; B: acetonitrile containing 0.1% (v/v) formic acid; C: isopropanol containing 0.1% (v/v) formic acid; D: ethanol containing 0.1% (v/v) formic acid; E: sample dispersed and dissolved by adding 2 mL tetrahydrofuran, then extracted with ethanol containing 0.1% (v/v) formic acid; F: methanol-tetrahydrofuran-water-perchloric acid (250:450:300:0.25, v/v/v/v) solution^[20]^. * Formula value: 4.00%, ND: not detected.

从表1我们可以看出,采用《规范》中的标准方法提取某乳液,亚甲基双-苯并三唑基四甲基丁基酚、双-乙基己氧苯酚甲氧苯基三嗪和甲酚曲唑三硅氧烷等脂溶性成分提取不完全。实验中发现,样品不能完全溶散,比色管刻度线上方分散部分样品,底部样品颗粒较大(见图2)。

**图 2:**
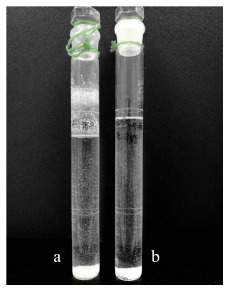
不同提取溶剂提取乳液的效果比较

因此,不能反映样品的真实情况。本研究采用0.1%(v/v)甲酸乙醇溶剂可以提取绝大部分防晒剂,但也存在上述部分脂溶性成分提取不完全的情况,因此先加入2 mL四氢呋喃溶散霜、乳等非蜡质样品,再用0.1%(v/v)甲酸甲醇溶剂提取可以获得满意结果,提取方法优于标准方法。

对于唇膏等蜡质化妆品,仅靠四氢呋喃涡旋振荡不能使其完全分散,检测结果偏低,而采用50 ℃超声20 min,唇膏样品分散完全,目标物充分溶出,测定结果准确。

### 2.3 线性范围、检出限及定量限

在优化的色谱条件下,分别对系列质量浓度的混合标准工作溶液进行测定,以各化合物的质量浓度为横坐标、色谱峰面积为纵坐标进行线性回归分析。结果显示,22种防晒剂在进样范围内,线性关系良好,相关系数(*r*)均大于0.998;向不含目标物的化妆品基质中添加不同质量浓度的标准溶液,按实验方法检测,以3倍信噪比(*S/N*≥3)时的目标物含量为检出限,以10倍信噪比(*S/N*≥10)时目标物含量为定量限,结果如表2所示。

**表 2:** 22种防晒剂的保留时间、线性范围、线性方程、相关系数、检出限及定量限

Analyte	*t*_R_/min	Linear range/(mg/L)	Linear equation	*r*	LOD/(mg/kg)	LOQ/(mg/kg)
PBS	3.86	0.96-47.98	*Y*=0.7549*X*+0.2661	0.9994	1.0	3.4
TDS	5.64	4.27-213.3	*Y*=0.0337*X*+0.0549	0.9993	21	70
BZ4	6.12	1.55-77.50	*Y*=0.3277*X*+0.2211	0.9995	2.4	8.0
CBM	7.36	0.91-45.53	*Y*=0.2168*X*+0.0770	0.9995	3.6	12
BZ2	7.78	2.53-126.5	*Y*=0.4771*X*+0.5320	0.9995	1.6	5.5
BCS	8.19	0.98-48.91	*Y*=0.5801*X*+0.2979	0.9996	1.3	4.2
BZ3	11.89	1.80-89.98	*Y*=0.4263*X*+0.3475	0.9996	1.9	6.3
3BC	14.60	0.87-43.75	*Y*=0.5434*X*+0.2126	0.9995	1.2	4.1
IMC	15.00	0.82-41.07	*Y*=0.9944*X*+0.3719	0.9995	0.7	2.4
MBC	16.47	1.11-55.59	*Y*=0.8400*X*+0.4407	0.9995	1.1	3.8
DHHB	18.03	2.06-102.9	*Y*=0.1141*X*+0.1106	0.9995	34	113
OCR	20.56	1.57-78.48	*Y*=0.3516*X*+0.2509	0.9995	13	43
BDM	21.13	2.48-124.0	*Y*=0.2109*X*+0.1761	0.9995	27	90
EDP	21.75	1.48-74.00	*Y*=1.0190*X*+0.7038	0.9995	5.0	17
EMC	23.37	0.94-46.96	*Y*=0.8941*X*+0.4590	0.9995	6.0	20
ES	25.32	2.48-123.9	*Y*=0.1669*X*+0.1235	0.9995	37	125
HS	26.51	2.34-116.8	*Y*=0.1518*X*+0.1988	0.9996	64	215
DBT	29.81	0.96-48.24	*Y*=1.4604*X*+0.6628	0.9995	2.9	9.7
ET	31.30	0.96-48.25	*Y*=1.6249*X*+0.5020	0.9992	2.7	8.9
DRT	31.61	1.85-92.51	*Y*=0.1754*X*+0.1493	0.9995	27	91
MBP	33.02	1.60-80.03	*Y*=0.5108*X*+0.3496	0.9995	2.0	6.6
EMT	33.64	0.69-34.56	*Y*=1.1356*X*+0.0976	0.9985	0.9	3.2

*Y*: peak area; *X*: mass concentration, mg/L.

### 2.4 回收率和精密度

向霜(不含蜡质)和唇膏(含蜡质)阴性样品中添加低、中、高3个水平的目标物标准溶液,依本研究建立的方法进行样品处理和测定,用标准曲线定量,计算方法的回收率,结果见表3。不同基质中各组分的加样回收率为85.2%~112.3%,相对标准偏差为0.5%~6.5%,表明该方法具有良好的准确性和精密度。

**表 3:** 空白化妆品中22种防晒剂的加标回收率和精密度(*n*=6)

Analyte	Spiked/ (g/kg)	Recoveries/%		RSDs/%		Analyte	Spiked/ (g/kg)	Recoveries/%		RSDs/%	
		Cream	Lip balm	Cream	Lip balm			Cream	Lip balm	Cream	Lip balm
PBS	1.9	98.5	89.9	2.3	4.4	OCR	3.1	96.5	99.0	2.4	2.5
	9.6	101.9	103.2	1.2	2.3		15.7	101.0	97.7	1.2	3.5
	48.0	108.0	102.0	0.8	2.2		78.5	106.7	97.2	2.1	1.2
TDS	8.5	97.6	94.1	3.8	1.7	BDM	5.0	92.5	105.0	3.6	3.4
	42.7	96.5	95.9	1.3	2.6		24.8	86.9	90.7	2.3	3.8
	213.3	101.5	100.4	1.0	2.0		124.0	91.9	85.2	1.5	3.1
BZ4	3.1	102.3	103.8	4.0	0.9	EDP	3.0	103.1	106.3	1.4	2.7
	15.5	94.9	101.1	0.9	2.1		14.8	96.9	102.6	2.3	4.4
	77.5	100.6	99.7	1.6	0.6		74.0	102.6	102.5	1.5	3.2
CBM	1.8	100.8	104.8	2.1	2.4	EMC	1.9	100.9	104.2	6.5	1.2
	9.1	95.8	102.0	1.5	3.2		9.4	104.2	101.6	1.9	2.7
	45.5	101.4	99.9	1.3	2.2		47.0	109.8	101.6	1.5	2.1
BZ2	5.1	102.6	109.0	3.1	0.8	ES	5.0	107.8	109.3	3.6	2.8
	25.3	94.3	101.4	0.7	3.1		24.8	102.8	101.0	1.1	1.6
	126.5	101.5	101.4	1.8	1.4		123.9	108.0	103.2	1.7	2.2
BCS	2.0	97.2	102.1	2.2	1.2	HS	4.7	100.2	103.0	1.2	3.6
	9.8	112.3	97.0	2.0	4.1		23.4	102.9	100.6	0.8	3.4
	48.9	104.9	96.8	4.2	2.1		116.8	108.2	101.7	1.5	3.3
BZ3	3.6	103.1	105.5	0.5	4.1	DBT	1.9	99.8	98.1	3.3	1.2
	18.0	102.3	100.6	1.8	3.8		9.6	94.6	100.5	3.3	2.1
	90.0	107.8	100.5	6.3	3.1		48.2	106.7	99.9	1.0	2.3
3BC	1.7	106.7	109.2	6.3	2.4	ET	1.9	94.9	105.8	3.2	1.3
	8.7	98.4	105.6	1.4	2.1		9.6	94.8	95.4	2.3	4.0
	43.7	103.5	105.2	2.8	3.1		48.2	103.4	94.1	1.7	2.3
IMC	1.6	103.0	106.4	1.5	3.3	DRT	3.7	101.7	103.2	2.3	2.5
	8.2	102.3	103.0	1.5	4.1		18.5	96.8	101.7	1.9	2.4
	41.1	108.2	103.0	4.5	2.6		92.5	102.6	98.4	2.4	2.3
MBC	2.2	102.7	104.8	2.0	3.0	MBP	3.2	91.8	94.2	3.7	1.1
	11.1	102.5	102.6	1.4	3.1		16.0	105.4	91.6	3.3	3.1
	55.6	107.7	102.5	2.8	2.7		80.0	102.3	91.6	1.7	2.4
DHHB	4.1	100.0	101.4	4.3	1.5	EMT	1.4	100.8	103.7	3.5	2.9
	20.6	100.9	97.4	1.5	1.1		6.9	98.5	105.7	3.4	2.0
	102.9	106.0	97.6	3.4	1.1		34.6	102.2	102.6	2.0	1.2

### 2.5 稳定性

《规范》使用的四氢呋喃-甲醇-水-高氯酸(450:250:300:0.25, v/v/v/v)溶剂^[20]^含有45%(v/v)四氢呋喃,沸点低,易挥发,混合储备溶液(100 mg/L)不能久置。-20 ℃条件下,储备液放置半个月,大部分组分浓度升高7%以上;苯基苯并咪唑磺酸组分易分解成两个相邻色谱峰。混合标准溶液(50 mg/L)室温放置12 h,甲酚曲唑三硅氧烷降解71%。

常温条件下,分别用甲醇配制高(50 mg/L)、中(10 mg/L)、低(2 mg/L)3个水平的混合标准溶液,于0、4、8、12、16、24、36、48、72 h各测定一次,计算每次测定的各组分峰面积的RSD。结果显示,除BDM、CBM和DRT外,其余19种防晒剂在72 h内均稳定,RSD均小于3.6%。低浓度条件下,BDM组分48 h内稳定(降解率<9.2%), CBM组分36 h内稳定(降解率<11%);中浓度条件下,CBM组分36 h内稳定(降解率<8.8%), DRT组分12 h内稳定(降解率<8.8%);高浓度条件下,DRT组分12 h内稳定(降解率<9.0%)。因此,样品处理后应一天内完成测定(除样品标签提示含有DRT需12 h内测定外),从而保证结果准确性。

用甲醇配制中、低浓度的DRT单标准储备液,72 h内均稳定,峰面积的RSD<1.0%。综上所述,溶剂中含有高氯酸(《规范》溶剂引入)或氢氧化钠溶液(配制PBS储备液引入),均会促使DRT降解发生。

### 2.6 实际样品的测定

用所建立的方法对市售的16份化妆品样品(其中乳6份、霜5份、露或液3份、喷雾1份、唇膏1份)进行防晒剂含量分析,并将测定的组分结果与产品标签和批件进行比对,结果见表4,部分样品的色谱图见图3。测定结果显示,所有样品检出的组分与产品标签和批件标示标识一致,表明产品稳定性良好,企业按批件配方生产。同时,“乳-1”“乳-2”“霜-1”“霜-2”和“霜-3”样品使用本方法测定的结果与厂家提供的配方值匹配良好。因此,该方法对不同基质的化妆品具有良好的适用性。

**表 4:** 市售化妆品样品中防晒剂的测定结果

Sample	Analyte	Content±*s*/ %	Formula value/%	Sample	Analyte	Content±*s*/ %	Formula value/%	Sample	Analyte	Content±*s*/ %	Formula value/%
Emulsion-1	MBC	2.06±0.01	2.00	Cream-1	IMC	2.80±0.03	3.00	Lotion-1	DHHB	2.46±0.06	-
	DHHB	2.30±0.01	2.50		OCR	1.02±0.01	1.00		EMC	5.84±0.15	-
	EMC	5.97±0.01	6.00		BDM	3.93±0.04	4.00		ET	0.75±0.02	-
	ET	1.72±0.01	1.70		EMC	6.96±0.05	7.00		DMT	4.53±0.11	-
	MBP	2.09±0.02	2.00		ES	4.13±0.04	4.00		MBP	0.72±0.02	-
Emulsion-2	OCR	7.74±0.05	8.00		MBP	1.21±0.02	1.20		EMT	2.29±0.06	-
	EMC	4.87±0.03	5.00		EMT	3.76±0.04	4.00	Lotion-2	DHHB	0.67±0.01	-
	HS	6.79±0.04	7.00	Cream-2	BZ3	3.95±0.02	4.00		OCR	2.83±0.01	-
	MBP	2.38±0.02	2.50		BDM	2.50±0.02	2.50		EMC	8.57±0.05	-
	EMT	3.34±0.03	3.50		EMC	8.22±0.03	8.00		ET	0.90±0.01	-
Emulsion-3	TDS	9.73±0.05	-		ES	2.88±0.02	3.00		MBP	0.76±0.01	-
	EMC	6.24±0.13	-	Cream-3	PBS	1.91±0.05	2.00		EMT	0.17±0.01	-
	DRT	4.86±0.09	-		DHHB	2.71±0.02	3.00	Lotion-3	DHHB	1.79±0.01	-
Emulsion-4	TDS	9.90±0.06	-		EMC	9.15±0.02	9.00		EMC	7.04±0.03	-
	DHHB	0.40±0.01	-		ET	0.82±0.01	0.85		ES	4.09±0.02	-
	EMC	6.12±0.06	-		MBP	1.96±0.01	2.00		EMT	2.11±0.05	-
	DMT	6.48±0.07	-		EMT	1.68±0.01	1.80	Spray	DHHB	3.33±0.05	-
	EMT	0.43±0.01	-	Cream-4	MBC	2.90±0.06	-		OCR	3.98±0.06	-
Emulsion-5	EMC	8.85±0.06	-		OCR	2.80±0.06	-		EMC	6.51±0.09	-
	ES	2.68±0.02	-		BDM	4.03±0.06	-		HS	2.57±0.03	-
	HS	0.97±0.01	-		EMC	7.31±0.15	-		ET	1.68±0.06	-
	MBP	2.26±0.02	-		ES	0.89±0.02	-		MBP	1.22±0.06	-
Emulsion-6	PBS	1.91±0.01	-		EMT	1.21±0.02	-		EMT	0.84±0.01	-
	BZ3	0.46±0.01	-	Cream-5	PBS	1.80±0.06	-	Lip balm	EMC	7.12±0.02	-
	OCR	3.75±0.01	-		MBC	1.93±0.03	-		ES	4.95±0.01	-
	BDM	2.54±0.03	-		OCR	4.56±0.06	-				
	ES	4.01±0.01	-		EMC	3.65±0.05	-				
	HS	4.19±0.01	-		EMT	0.82±0.01	-				
	MBP	0.75±0.01	-								
	EMT	0.82±0.01	-								

*s*: standard deviation; -: not available.

**图 3:**
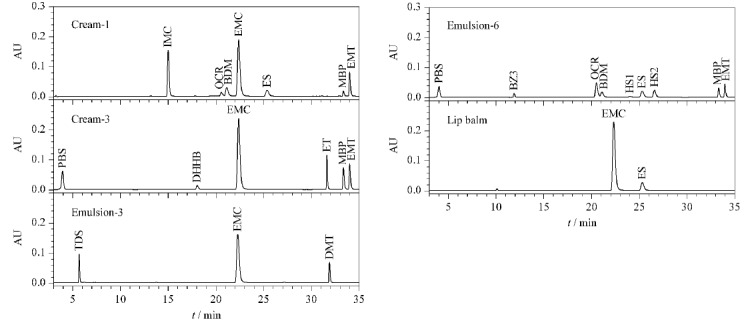
部分市售化妆品的液相色谱图

## 3 结论

本研究在《化妆品安全技术规范》(2015年版)的基础上,分别就色谱柱种类、流动相组成、样品提取溶剂选择等方面进行了优化,使用乙腈-异丙醇-50 mmol/L乙酸铵水溶液(含0.05%(v/v)甲酸)梯度洗脱,可测定化妆水、乳液、膏霜、唇膏等化妆品中的22种防晒剂。与原方法比较,流动相摒弃了四氢呋喃和高氯酸等高腐蚀性溶剂,增加了被测组分二苯酮-2。被测物稳定性更好,实验操作更为便利高效。实验结果表明,本研究方法快速简便、重现性和耐用性好,结果准确可靠,兼顾绿色环保,有助于防晒类化妆品的质量监控。

## References

[1] Salvador A, Chisvert A. Anal Chim Acta, 2005,537:1

[2] Regulation (EC) No.1223/2009 of the European Parliament and of the Council of 30 November 2009 on Cosmetic Products. [2009-11-30]. http://eur-lex.europa.eu/legal-content/EN/TXT/?uri=celex%3A32009R1223http://eur-lex.europa.eu/legal-content/EN/TXT/?uri=celex%3A32009R1223

[3] National Medical Products Administration. No.268/2015 Bulletin of the State Administration for Food and Drug of the People’s Republic of China.[2015-12-23]. https://www.nmpa.gov.cn/hzhp/hzhpggtg/hzhpqtgg/20151223120001986.htmlhttps://www.nmpa.gov.cn/hzhp/hzhpggtg/hzhpqtgg/20151223120001986.html

[4] Meng X S, Ma Q, Bai H, et al. Chinese Journal of Chromatography, 2015,33(8):799 26749854 10.3724/sp.j.1123.2015.03024

[5] Feng L, Zhao Y X, Wang J L, et al. Chinese Journal of Health Laboratory Technology, 2018,28(7):789

[6] Tu X Q, Yang L, Meng Q Y, et al. Flavour Fragrance Cosmetics, 2020(1):55

[7] Han X P, Zhou P, Li Y N. Chinese Journal of Health Laboratory Technology, 2018,28(9):1032

[8] Sun J, Cao L, Feng Y L, et al. Physical Testing and Chemical Analysis (Part B: Chemical Analysis), 2019,55(10):1186

[9] Liu X, Gong H. Detergent & Cosmetics, 2018,41(6):46

[10] Cui F J, Gu J, Zhang K, et al. China Surfactant Detergent & Cosmetics, 2017,47(1):52

[11] Guo J, Liu Q, Liu C H, et al. Physical Testing and Chemical Analysis (Part B: Chemical Analysis), 2020,56(4):400

[12] Xu L, Pang D B, Fu L M, et al. Physical Testing and Chemical Analysis (Part B: Chemical Analysis), 2015,51(12):1666

[13] Maria A K, Eric L, Eric C, et al. Anal Chim Acta, 2018,1034:184 30193633

[14] He Y L, Wang Z, Yan X H. Chinese Journal of Health Laboratory Technology, 2017,27(22):3221

[15] Bian H T, Mao X Q, Qu B C. China Surfactant Detergent & Cosmetics, 2013,43(6):477

[16] Lin W X, Sun X Q, Ma J. Chinese Journal of Chromatography, 2013,30(5):410 10.3724/sp.j.1123.2012.11015 24010338

[17] Gao L, Wang Y Q, Liu Y, et al. Yunnan Chemical Technology, 2019,46(12):109

[18] Ding Y R, Huang Y, Zhao T T, et al. Chinese Journal of Chromatography, 2014,32(6):629 25269262 10.3724/sp.j.1123.2014.01035

[19] Zhou B L. Physical Testing and Chemical Analysis (Part B: Chemical Analysis), 2019,55(9):1013

[20] National Medical Products Administration. No. 40/2019 Bulletin of the National Medical Products Administration of the People’s Republic of China.[2019-07-05]. http://www.nmpa.gov.cn/xxgk/ggtg/qtggtg/20190711165901174.htmlhttp://www.nmpa.gov.cn/xxgk/ggtg/qtggtg/20190711165901174.html

